# Optimizing transformations for automated, high throughput analysis of flow cytometry data

**DOI:** 10.1186/1471-2105-11-546

**Published:** 2010-11-04

**Authors:** Greg Finak, Juan-Manuel Perez, Andrew Weng, Raphael Gottardo

**Affiliations:** 1 Vaccine and Infectious Disease Division, Fred Hutchinson Cancer Research Center, 1100 Fariview Ave N, Seattle, WA, 98109, USA; 2 Computational Biology Unit, Institut de Recherches Cliniques de Montréal, 110 Pine Ave West, Montreal, QC, H2W 1R7, Canada; 3 Terry Fox Laboratory, 675 West 10th Avenue Vancouver, BC, V5Z 1L3, Canada

## Abstract

**Background:**

In a high throughput setting, effective flow cytometry data analysis depends heavily on proper data preprocessing. While usual preprocessing steps of quality assessment, outlier removal, normalization, and gating have received considerable scrutiny from the community, the influence of data transformation on the output of high throughput analysis has been largely overlooked. Flow cytometry measurements can vary over several orders of magnitude, cell populations can have variances that depend on their mean fluorescence intensities, and may exhibit heavily-skewed distributions. Consequently, the choice of data transformation can influence the output of automated gating. An appropriate data transformation aids in data visualization and gating of cell populations across the range of data. Experience shows that the choice of transformation is data specific. Our goal here is to compare the performance of different transformations applied to flow cytometry data in the context of automated gating in a high throughput, fully automated setting. We examine the most common transformations used in flow cytometry, including the generalized hyperbolic arcsine, biexponential, linlog, and generalized Box-Cox, all within the BioConductor *flowCore *framework that is widely used in high throughput, automated flow cytometry data analysis. All of these transformations have adjustable parameters whose effects upon the data are non-intuitive for most users. By making some modelling assumptions about the transformed data, we develop maximum likelihood criteria to optimize parameter choice for these different transformations.

**Results:**

We compare the performance of parameter-optimized and default-parameter (in *flowCore*) data transformations on real and simulated data by measuring the variation in the locations of cell populations across samples, discovered via automated gating in both the scatter and fluorescence channels. We find that parameter-optimized transformations improve visualization, reduce variability in the location of discovered cell populations across samples, and decrease the misclassification (mis-gating) of individual events when compared to default-parameter counterparts.

**Conclusions:**

Our results indicate that the preferred transformation for fluorescence channels is a parameter- optimized biexponential or generalized Box-Cox, in accordance with current best practices. Interestingly, for populations in the scatter channels, we find that the optimized hyperbolic arcsine may be a better choice in a high-throughput setting than current standard practice of no transformation. However, generally speaking, the choice of transformation remains data-dependent. We have implemented our algorithm in the BioConductor package, flowTrans, which is publicly available.

## Background

Flow cytometry (FCM) is increasingly moving to-wards automated methods to deal with the quantities of data generated by high throughput, high-content screening [1-11]. An appropriate, auto-mated data pre-processing pipeline, including automated gating and matching of corresponding cell populations across replicated or similar samples is important for the accuracy of downstream analysis. However, accurate automated gating of FCM data is complicated by asymmetric and overlapping cell populations, frequent outlier events, cell populations whose variance depend on their mean fluorescence intensity, and multiplicative errors in the fluorescence channels. All of these characteristics can influence the output of both manual and automated gating, and subsequent downstream analysis. Data transformation plays an important role in mitigating some of these effects, both in manual and automated analysis setting. In a manual analysis setting, a transformation is typically chosen to facilitate cell population visualization for the purposes of gating. Generally, a set of common transformation parameters are chosen across multiple samples to ensure that they are on a common scale and facilitate comparison. Carefully chosen data transformations and corresponding parameters have been suggested to overcome some of the problems surrounding manual FCM analysis and gating [12,13]. Data transformation plays an even more important role in an automated, high throughput setting since the scale and distribution of the transformed data can influence downstream analysis procedures. Some automated gating methods include data transformation as part of the gating algorithm [6,8,10]. Other methods function under the assumption that the data have been appropriately transformed prior to the gating step [1,2,7,9]. Methods for inter-sample normalization in downstream preprocessing steps can allow for different data transformations per sample. By loosening the requirements of a common transformation across samples, we can explore the optimization of data transformations for automated gating. In such a setting, the impact of data transformation has received relatively little attention [6,14,15]. Lo *et al*. propose the estimation of a generalized Box-Cox transformation embedded within a mixture modelling framework to simultaneously gate and transform skewed cell populations [6]. Their approach works very well in practice, but FCM data are generally still subjected to a global transformation prior to automated gating. What is clear is that the choice of transformation is data-driven and involves multiple considerations. The underpinning principle is to choose a transformation that facilitates cell population gating, visualization, and inter-sample comparison, by obtaining a representation wherein cell populations are well resolved across the full range of the data [6,12,15-17].

There are many transformations in common use for flow cytometry data, including the logarithm and related transformations such as the linear-logarithmic and hyperlog transforms, power transformations such as the generalized Box-Cox, which includes the logarithm as a special case, and the biexponential and related transformations such as the logicle and generalized arcsinh [6,12,13,17]. The log transformation can often stabilize the variance of cell populations in the fluorescence channels across nearly the full range of intensities but cannot represent negative data values of unstained cell populations, leading to compression of data against the axes and poor visual representation of low intensity or unstained populations [12]. To deal with this, other transformations have been suggested, including the linear-logarithmic (linlog) transformation, the biexponential (logicle), and generalized arcsinh transformations. These all improve upon the log by allowing negative values, providing a linear representation of data around zero and a logarithmic representation of the data at higher intensity values, with a smooth transition between the two extremes. The hyperlog has been proposed specifically for compensated data and is also capable of dealing with non-positive values [13]. The biexponential transform provides additional flexibility by allowing the linear portion of the scale to be asymmetric around zero; the logarithmic scale can similarly be tuned by adjustable parameters. The generalized Box-Cox has been adapted and applied to FCM data in the context of automated gating within a multivariate-*t *mixture modelling framework [4,6,18]. Lo *et al*. proposed to select the generalized Box-Cox transformation parameter maximizing the likelihood of individual cell populations being generated by a mixture of multivariate-*t *or multivariate-normal distributions on the transformed scale [6]. All of these transformations have one or more parameters (with the exception of the log) that can be adjusted in a data-dependent manner to modify the representation of the data. We perform our analysis within the *flowCore *framework in BioConductor, which is the predominant tool set in use for automated, high throughput flow data analysis. The default parameters of the transformations within *flowCore *are rarely adjusted in practice, and are almost certainly not the best possible parameter choices for all data sets. Here we examine the impact of the chosen transformation and its parameters upon the accuracy of automated gating as well as the ability to match gated cell populations across samples.

We make several important comments about notation. In this paper we refer frequently to transformations and inverse transformations. Mathematically, a function and its inverse are denoted *f*(.) and *f *^-1^(.), respectively. However, in FCM data, the biexponential *transformation *is actually the inverse of *f*(*y*) = *a *exp(*b*(*y *- *w*)) - *c *exp(-*d*(*y *- *w*)) + *f*, where *f*(*y*) is the biexponential *function*. For clarity of exposition, in this paper, we will refer to the biexponential transformation as *f *^-1^(*y*) above, and the inverse-biexponential transformation as *f*(*y*), above. Although counter-intuitive to the mathematical definition of the biexponential *function*, this nomenclature is accepted in the FCM community [12]. The notation in Table 1 is in accordance with this nomenclature.

**Table 1:** Summary of transformations

Transformation	Mathematical Definition *f*(*y*;*θ*), *f*^-1 ^(*x*; *θ*)	Jacobian *J_θ _*(*y*)	Parameter Bounds and Constraints
Linlog	$\begin{array}{l} f(y;\theta )=\left\{ \begin{array}{l} (y-\theta )/\theta +\log(\theta );y\leq \theta \\ \log(y);y>\theta \end{array}\right.\\ f^{-1}(x;\theta )=\left\{ \begin{array}{l} \theta (x-\log\theta +1);x<\log(\theta )\\ \exp(x);x\geq \log(\theta ) \end{array}\right. \end{array}$	$\begin{array}{l} 1/\theta ;\;y\leq \theta \\ 1/y;\;y>\theta \end{array}$	$\begin{array}{l} \theta \in [\min(y),\max(y)],\\ \theta \geq 0 \end{array}$

Generalized Arcsinh	$\begin{array}{l} f(y;\theta )=\log(a+by+\sqrt{{\left(a+by\right)}^{2}+1})+c\\ f^{-1}(x;\theta )=\frac{1}{2}\left(e^{\left(x-c\right)}-e^{-(x-c)}\right) \end{array}$	$\frac{b+\frac{1}{2}(2(ba+b^{2}y){\left({\left(a+by\right)}^{2}+1\right)}^{-1/2})}{a+by+\sqrt{{\left(a+by\right)}^{2}+1}}$	$\begin{array}{l} \theta =\{a,b,c\};a,c\geq 0;\\ b>0 \end{array}$

Biexponential	$\begin{array}{l} f(y;\theta )=\text{no closed form}\\ f^{-1}(x;\theta )=ae^{\left(b(x-w)\right)}-ce^{\left(-d(x-w)\right)}+\text{f} \end{array}$	1 = (*abe*^*b*(*x-w*)^+ *cde*^-*d*(*x-w*)^)	$\begin{array}{l} \theta =\{a,b,c,d,\text{f},w\};a,c\in (0,1]\\ \text{f}=0,w\in \mathbb{R},b,d\geq 0 \end{array}$

Generalized Box-Cox	$\begin{array}{l} f(y;\theta )=\begin{array}{c} \frac{\mathrm{sgn}(y)|y{|}^{\theta }-1}{\theta };\theta \in \mathbb{R} \end{array}\\ f^{-1}(x;\theta )=\begin{array}{c} \mathrm{sgn}(\theta x+1)|\theta x+1{|}^{\frac{1}{\theta }};\theta \in \mathbb{R} \end{array} \end{array}$	|*y*|^*θ*-1^	*θ *∈ ℝ

Summary of transformations for flow cytometry. The transformations examined in this study, together with their inverses, Jacobians and parameter restrictions. *f*(*y*; ***θ***) is the transformation function typicallly applied to untransformed flow cytometry data, y, whereas *f*^-1^(*x*; ***θ***) is its inverse. For the biexponential, the transformation *f*(.) has no closed form and must be solved numerically. Consequently, the Jacobian of the biexponential transformation is given by the reciprocal of the Jacobian of the inverse transformation, and therefore depends directly on the transformed data, *x*. sgn is the signum function, also known as the sign function, which extracts the sign of a real number.

## Methods

### Parameter Estimation

We use maximum likelihood methods to estimate the parameters of each transformation (generalized arcsinh, generalized Box-Cox, linlog, and biexponential) following established methods [19]. If **Y **is an *n *× *d *data matrix, we represent the data as a sequence of *n d*-dimensional vectors **Y***_i _*={*y*_*i*, 1_,..., *y_i, d_*} and the transformed data as $Y_{i}^{\left(\theta \right)}=f(y_{i}|\theta )$, where *f *is the transformation function, and ***θ ***is the vector of transformation parameters. For simplicity we model the transformed data by a multivariate normal distribution **Y**^(***θ***) ^~ *N_d_*(***μ***, **Σ**|***θ***). The likelihood of the parameters, given the data and a fixed ***θ ***is(1)

where $J_{\theta }(Y_{i})=|\frac{\partial (y_{i,1}^{\left(\theta \right)},\ldots ,y_{i,d}^{\left(\theta \right)})}{\partial (y_{i,1},\ldots ,y_{i,d})}|$ is the Jacobian term that accounts for the change of scale under different transformation parameters. It follows that for a fixed ***θ***, the mean and covariance of the transformed data can be estimated by the sample mean and sample covariance, as follows,

$$\begin{array}{l} \hat{\mu }=\frac{1}{n}\sum_{i=1}^{n}Y_{i}^{\left(\theta \right)}=\bar{Y^{\left(\theta \right)}}\\ \hat{\Sigma }=\frac{1}{n}\sum_{i}(Y_{i}^{\left(\theta \right)}-\bar{Y^{\left(\theta \right)}}){\left(Y_{i}^{\left(\theta \right)}-\bar{Y^{\left(\theta \right)}}\right)}^{T} \end{array}$$

Now we can substitute these two expressions into (1) and maximize over ***θ ***After some simple algebra, it can be shown that it is equivalent to minimizing:

$$S(\theta )=\frac{|(\sum_{i}\left(Y_{i}^{\left(\theta \right)}){\left(Y_{i}^{\left(\theta \right)}\right)}^{T}/n-(\bar{Y_{i}^{\left(\theta \right)}}){\left(\bar{Y_{i}^{\left(\theta \right)}}\right)}^{T})\right|}{G(\theta )}$$

where $G(\theta )={\left(\prod_{i}J_{\theta }{\left(Y_{i}\right)}^{2}\right)}^{1/n}$ is the geometric mean of the squared Jacobian terms. Thus explicit derivation of the functions to be minimized for each transformation, **S**(***θ***), only involve deriving the Jacobian of the transformation. These transformations, their inverses, and their Jacobians are summarized in Table 1, and presented in Additional File 1 with common parameter values. When no closed form exists, we use numerical optimization routines in R to optimize (1) over ***θ ***[20]. We note that the translation parameter is fixed at *f *= 0 in the biexponential transformation in order to resolve identifiability issues with the full parameterization (see Additional File 2). The decision to model the *transformed *data as a multivariate Gaussian distribution is motivated by the implicit assumption of a common error model across all cells when performing a global data transformation. We note that the form of this implicit error model is not known. The multivariate Gaussian assumption is strictly a computationally convenient choice that has the effect of making the marginal distribution of the data more symmetric and reducing the influence of outliers at the subsequent gating step. For the purposes of gating, however, flow cytometry data is better represented as a mixture of distributions, which is done explicitly by *flowClust *and *flowMerge*.

### Follicular Lymphoma Data Set

We examined a subset of a clinical FCM data set derived from lymph node biopsies from 10 individuals diagnosed with follicular lymphoma. Each sample was five-dimensional, labeled with CD5, CD19, and CD3, in addition to the FSC (forward scatter) and SSC (side scatter) channels. The ten individuals sampled consisted of five males and five females, of median age 59.5 years, (range 40-82). Five individuals had stage 4 disease, three had stage 3 disease, and two had stage 1 disease. Overall survival for the group ranged from 0.45 years to 14.66 years.

### Evaluating the Suitability of a Data Transformation

To evaluate the performance of different transformations, we measured the variation in the location of cell populations identified by automated gating using the flowClust/flowMerge frameworks [4,6]. The variation in the position of gated cell populations was measured across biological replicates, and compared for each transformation.

**Figure 1:**
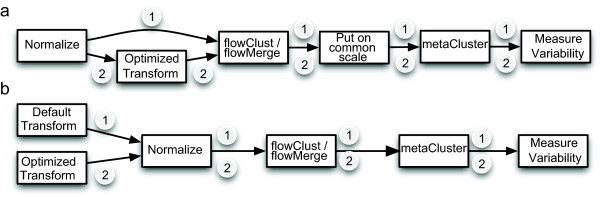
**Flowchart of the analysis pipeline**. Flowchart describing our analysis pipeline. a) Procedure for analyzing FSC vs SSC channels. Standard data analysis procedures are depicted by path 1, whereas procedures applying parameter-optimized transformations are depicted by path 2. b) Procedure for analyzing fluorescence channels. Standard procedures are depicted by path 1). Procedures utilizing optimized transformations are depicted by path 2. The default transformation depicted in 1) is the generalized arcsinh with default parameters (a = 1, b = 1, c = 0), as defined in the *flowCore *package. Normalization follows transformation in b) to ensure that the transformed data are on a common scale.

The FSC and SSC channels were analyzed separately from the fluorescence channels, according to procedures established elsewhere [6]. Data was normalized on the scale on which it would be visualized, therefore the scatter channels were normalized prior to transformation, whereas the fluorescence channels were normalized after data transformation (Figure 1a and 1b) [21]. In the scatter channels, parameter-optimized transformations (generalized arcsinh, generalized Box-Cox, linlog, biexponential) were compared against default-parameter counterparts (generalized arcsinh and biexponential), as well as the standard procedure of no transformation (Figure 1a path 1 and 2). For fluorescence channels, default and optimized parameter transformation were compared against together with the log transformation (Figure 1b, path 1 and 2). Populations in the scatter and fluorescence channels were automatically gated using the *flowClust *and *flowMerge *framework [4,6]. These discovered populations were metaclustered, and intracluster variability was compared either on the transformed scale (fluorescence channels), or on the original scale (scatter channels).

### Cell Population Identification, Metaclustering and Metrics

Automated gating was performed using the *flowClust *and *flowMerge *packages in BioConductor [4,6,22]. Lymphocyte cell populations were identified manually from among the gated populations, and corresponded to the most dense clusters of cells in the FSC vs SSC dimensions. We evaluated the performance of each transformation by examining the variation in the position of the lymphocyte cell populations across biological replicates. The variation was measured as the sum of squared deviations from the mean lymphocyte cell population position across the samples. Cell populations identified in the fluorescence channels by automated gating were matched across samples using a *metaclustering *approach. This involved a modified single linkage agglomerative clustering approach to group corresponding populations across samples [23]. We provide an informal description of the algorithm, followed by a formal definition. Informally, we assume that cell populations in each sample are unique, therefore no more than one population from a sample can belong to a metacluster. We choose the sample with the largest number of cell populations and assign each population to unique metacluster. We then iterate through each cell population in the remaining samples and assign it to its nearest metacluster (measured by the Mahalanobis distance), with the restriction that no more than one cell population from a sample may belong to the same metacluster.

More formally, we let *S *= {*s*_1_, . . , *s_n_*, . . , *s_N _*} be the set of all samples, indexed by *n*. Following automated gating, a sample is clustered into some number, π*_n_*, of *discovered cell populations*. We let $P^{n}=\{p_{1}^{n},..,p_{i}^{n},..,p_{\pi_{n}}^{n}\}$ be the set of discovered cell populations within the *n*th sample, such that the cardinality of *P^n ^*is |*P^n^*| = π*_n_*. On the transformed scale, each discovered cell population is summarized by a multivariate-*t *distribution. The parameters of the multivariate-t distribution representing the *i*th cell population from the *n*th sample are $\mu_{i}^{n}$, the *d*-dimensional vector of means, $\Sigma_{i}^{n}$, the *d *× *d *covariance matrix, and $\nu_{i}^{n}$, the degrees of freedom, as defined in Lo et. al. [6].

The set of metaclusters is denoted ℳ = {*M*_1_, *M*_2_, ..., *M_K_*}. Each metacluster is itself a set of populations with the constraint that no metacluster can contain more than one population from a given sample. For each population, $p_{i}^{n}$, we define a label $l_{i}^{n}$ which can take a value between 1 and *K*, where *K *= max*_n_*(*π_n_*). If $l_{i}^{n}$ = *k*, population $p_{i}^{n}$ belongs to metacluster *k*. Additionally the distance between a metacluster *M_k _*and a population $p_{j}^{m}$ is denoted $D(k,p_{j}^{m})=\min_{\left\{i,n|l_{i}^{n}=k\right\}}[D(p_{i}^{n},p_{j}^{m})]$, which is the minimum distance between population $p_{j}^{m}$ and all populations that are already assigned to metacluster *k*. To enforce the one sample per metacluster constraint we set $D(k,p_{j}^{m})=\infty$ if $l_{i}^{m}=k$ for some *m*. We define the distance between population *i *in the *n*th sample and population *j *in the *m*th sample as $D(p_{i}^{n},p_{j}^{m})=\sqrt{{\left(\mu_{i}^{n}-\mu_{j}^{m}\right)}^{T}\Sigma_{i}^{n-1}(\mu_{i}^{n}-\mu_{j}^{m})}$, which is the *Mahalanobis *distance, assuming that the first argument ($p_{i}^{n}$) is a population assigned to metacluster *k *[24].

To construct the metaclusters we:

1. Set the number of metaclusters to *K *= max*_n_*(π*_n_*)

2. Initialize the metaclusters with the *K *populations from sample $s_{n_{0}}$ where *n*_0 _= arg max*_n_*(π*_n_*). If there is more than one such sample, pick the one with the best likelihood or best separation of clusters (entropy).

3. Let $\left(j,n,k)=\arg\min_{\left\{j,n,k\right\}}D(k,p_{j}^{n}\right)$, assign $p_{j}^{n}$ to metacluster *k*.

4. Repeat until each population is assigned to a metacluster.

### Simulation Study

To further test our algorithm, we simulated ten data sets of *N *= 15000 events in three-dimensions from nine cell populations modeled as a multivariate-*t *mixture distribution with four degrees of freedom, following the approach of Lo *et al*., and fixed proportions drawn from a Dirichlet distribution with parameter *α *= (1, 1, 1, 1, 1, 1, 1, 1, 10) [6]. The population proportions were,

(2) $$\begin{array}{l} p=(0.0477,0.0351,0.0101,0.0678,\\ 0.0756,0.0730,0.1330,0.0677,0.490) \end{array}$$

**Figure 2:**
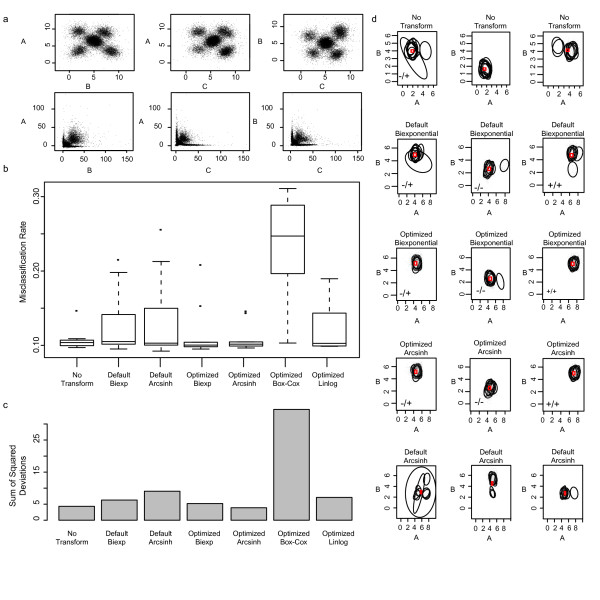
**Simulation study results**. Results of transformations on simulated data. a) A single simulated sample is shown as a series of bivariate dot plot projections. Data are presented on the original scale (top row), and on the scale of the inverse biexponential transform (bottom row). Points represent individual events. b) Boxplots representing the distribution of misclassification rates of *flowMerge *models with K = 9 components fitted to the simulated data set under different transformations. c) Intra-cluster variability measured as the total sum of squared deviations for metaclustered populations identified by *flowMerge *under different transformations. d) Example bivariate projections of metaclusters for untransformed data (top row), default biexponential (second row), optimized biexponential (third row), optimized generalized arcsinh (fourth row), and default generalized arcsinh (fifth row). Corresponding metaclusters were selected where possible. Metaclusters are labeled as +/+, -/-, -/+ for artificial markers A and B. Ellipses represent 90th quantile contours of subpopulations.

Eight cell populations were distributed in three dimensional space at the eight corners of a cube, with one cell population located in the center of the cube (Figure 2a, top row). Real FCM data frequently contain one cell population that has higher density than the other cell populations in the mixture. We simulated this characteristic of FCM data by as-signing higher weight to the central population in our simulated data and it is reflected in the parameters of the Dirichlet. Simulated cell population locations ranged from zero to seven in arbitrary units, and corresponding populations has variance *σ*^2 ^= 0.25 across the ten data sets. The simulated data were transformed (Figure 2a, bottom row) by the inverse biexponential using different, randomly chosen transformation parameters for each sample ({*a*, *c*} ~ *U*(0, 1) and {*b*, *d*} *U*(0, 2)), where {*X*, *Y*} ~ *U*(*p*, *q*) denotes that variables *X *and *Y *are independently drawn from a Uniform distribution over the interval [*p*, *q*]. We applied our algorithm to this inverse-transformed data, optimizing transformation parameters for the generalized arcsinh, biexponential, generalized Box-Cox, and linlog transforms in order to recover **Y**^(***θ***)^. We then compared the output of these transformations against the default-parameter versions of the biexponential and generalized arcsinh transforms, as well as the original untransformed data. To assess the performance of different transformations the data were normalized, gated using flowClust/flowMerge, and the discovered populations were clustered across data sets (metaclustered) [21]. We then measured the resulting intra-metacluster variability as well as the misclassification rate for individual events in the discovered populations, relative to their true class membership (Figure 2b-d) [4].

## Results

### Follicular Lymphoma Data

**Figure 3:**
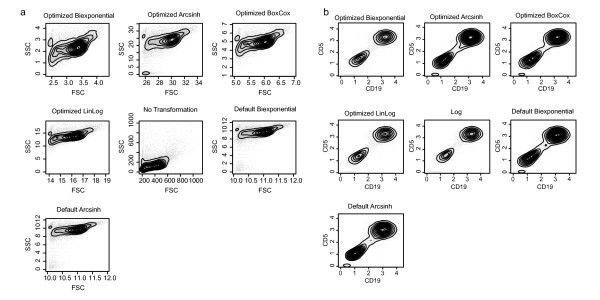
**Visualization of FCM data under different transformations**. Visualization of flow cytometry data under different transformations. a) The FSC and SSC dimensions of a representative sample transformed using the biexponential, generalized arcsinh, generalized Box-Cox, and linlog transformations with optimized parameters. Untransformed and default-parameter generalized arcsinh and biexponential transformed data are shown for comparison. Some parameter-optimized trans-formations (biexponential and generalized Box-Cox in this example) improve visualization and resolution of the lymphocyte cell population when compared to default or untransformed data. b) Comparison of a fluorescence channel data under different parameter optimized and default transformations. In this example, the optimized biexponential and optimized linlog improve the resolution of the two populations in the CD19 vs CD5 channels, compared to the default generalized arcsinh or default biexponential. Points represent individual events, contours represent the two-dimensional kernel density estimate of the data.

**Figure 4:**
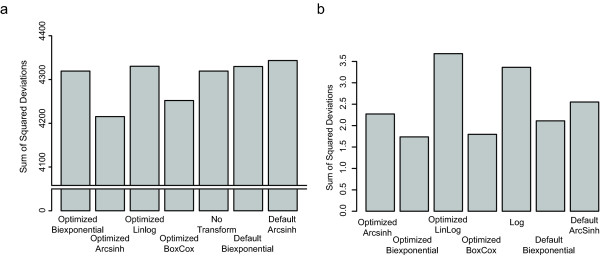
**Variation in cell subpopulation locations**. Variation in population location measured as the sum of squared distances between population centers for a) lymphocyte populations in the FSC vs SSC channels. b) metaclusters of cell populations in the fluorescence channels.

Our approach to data analysis of fluorescence and scatter data differs slightly, in that scatter data are normalized prior to transformation, while fluorescence data are normalized post-transformation, in accordance with common practice (Figure 1a-b). We examined the effects of parameter-optimized transformations compared to their default-parameter counterparts on visualization of cell populations in the scatter and fluorescence channels (Figure 3a, b). For scatter channels, differences between parameter-optimized, default, and untransformed data are clearly visible (Figure 3a). The optimized version of the biexponential, generalized arcsinh, and generalized Box-Cox, all provide improved visualization of cell populations than the default-parameter biexponential, generalized arcsinh, or the untransformed data. For the fluorescence channels, the data are put on a common scale following transformation (see Materials and Methods), and distinct differences can be seen between the optimized and default transformations (Figure 3b). Populations are better resolved following some transformations than others. The optimized biexponential improves visualization of cell populations compared to the biexponential with default parameters, while there is no observable difference between the default and optimized generalized arcsinh transformation. Other transformation show similarly variable results. Although differences between optimized and default transformations are subtle under visual inspection, these subtleties can have significant effects on model fitting and model selection during automated gating if they lead to violations of model assumptions (Figure 4a, b). For example, small deviations from symmetry in the shape of cell subpopulations can lead to selection of models with more components, more parameters, or different degrees of freedom, leading to different final gates.

To obtain a quantitative measure of the effect of different transformations on the automated analysis of real-world flow cytometry data, we proceeded to perform automated gating populations in the scatter and fluorescence channels, as described in the Methods. We measured the variability between discovered populations by metaclustering them across samples, and measuring the intra-metacluster variability as the sum of squared deviations (Figure 4a, b). There were minimal differences in the variability of the lymphocyte populations between different transformations applied to the scatter channels (Figure 4a). The optimized generalized arcsinh and optimized generalized Box-Cox transformations had the lowest variation in metaclusters, performing better than the default generalized arcsinh, or the standard of no transformation. In contrast, larger differences in variability were observed between transformations for populations in the fluorescence channels (Figure 4b). The optimized biexponential, optimized generalized Box-Cox, and default biexponential exhibited the lowest intra-metacluster variation, whereas the optimized linlog, log, and default generalized arcsinh exhibited the highest intra-metacluster variation. However, on the scale of the scatter data, it appears that the differences between transformations are not large.

**Figure 5:**
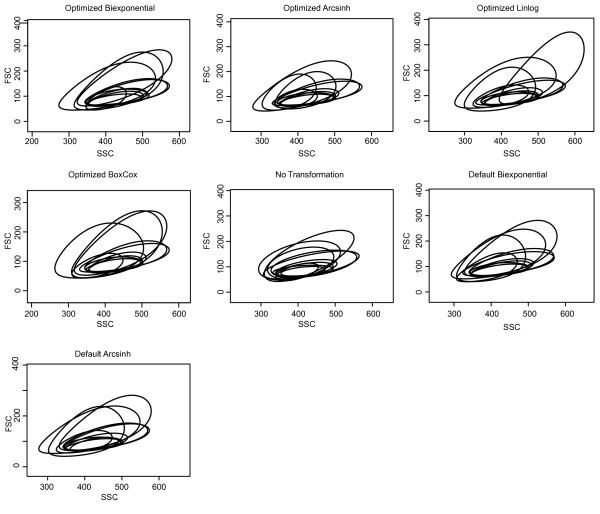
**Metaclusters in the scatter channels**. Metaclusters of lymphocyte populations in the FSC and SSC dimensions for four optimized and three default transformations. We observe little difference between the transformations in the forward and side scatter dimensions for gating lymphocyte populations, suggesting that the primary benefit in the FSC vs SSC dimensions is for data visualization. Contours represent the 90th quantiles of the cell subpopulation distributions.

**Figure 6:**
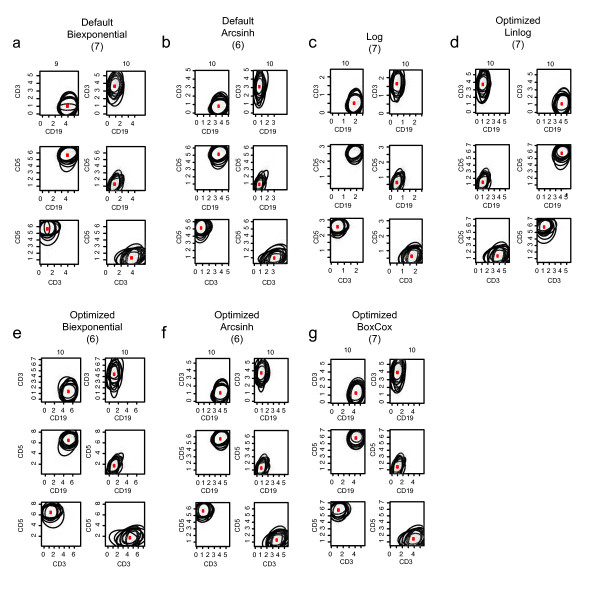
**Metaclusters in the fluorescence channels**. Metaclusters of cell populations defined in the fluorescence channels of the lymphoma data under different transformations. Only the primary cell populations are shown for comparison (CD19+/CD3-/CD5+ and CD19-/CD3+/CD5-). The number of metaclusters for the transformed data is shown in brackets. The number of cell populations in a metacluster is shown above the plot. Contours represent the 90th percentiles of the cell subpopulation distributions.

The differences between lymphocyte metaclusters in the scatter dimensions are readily seen to be minimal when visualizing the metaclusters directly on the original scale (Figure 5, contour show's 90th percentile of the population). Each metacluster contains all ten lymphocyte populations from the ten samples in the data set. Similarly, metaclusters in the fluorescence channels are visualized directly on the transformed scale (Figure 6a-g, contours show 90th percentile of the populations). The metaclusters of the primary cell populations in the samples are shown (CD3-/CD19+/CD5+ and CD3+/CD19-/CD5-), represented as a series of bivariate projections for each transformation examined. Interestingly, with the exception of the default biexponential transform, all metaclusters contain representative cell populations from all samples in the data set. Additionally, the total number of metaclusters varies between transformations (shown in brackets), indicating that the principal source of variation in the metaclustering is due to cell populations represented as outliers in the flowClust/flowMerge gating, rather than due differences in the well-defined, primary cell populations in the samples.

### Simulation

Simulated data allows access to the "true" class memberships of individual events that are not available with real-world data, and can help to better understand the effects of transformation and parameter selection on automated gating. We again briefly describe our approach here. We simulated ten data sets, transforming each with the inverse of the biexponential transform using randomly chosen parameter values, as described in the methods (Figure 2a). We then applied our algorithm to the inverse transformed data to estimate optimal parameters under different transformations and again transformed the inverse-transformed data using those estimated parameters and the appropriate transformation. The data output by this process, which should reflect the distribution of the original data, was subjected to automated gating. By comparing the true class membership of events in the original data against the class membership of events gated by flowClust/flowMerge, we computed the misclassification rate of the gating model under different optimized and default transformations (Figure 2b). When the optimized transformation was of the same family as the inverse transformation (i.e. generalized arcsinh or biexponential) we observed misclassification rates comparable to the rates obtained for the untransformed data (i.e. the gold standard data transformed using the correct transformation parameters) (mean misclassification rate of 10.7%, 10.0% and 10.1% for untransformed data, optimized biexponential and optimized generalized arcsinh, respectively) (Figure 2b). In contrast, the optimized generalized Box-Cox, optimized linlog, as well as the default generalized arcsinh and biexponential had considerably increased misclassification rates with higher variability across the simulated samples (mean misclassification rate of 23.3%, 11.8%, 12.7%, and 13.2% for optimized generalized Box-Cox, optimized linlog and default generalized arcsinh, default biexponential, respectively) (Figure 2b). The poor performance of the optimized generalized Box-Cox is not surprising in this case, since the biexponential inverse-transformation applied to the data was quite different from the generalized Box-Cox transform. This demonstrates that optimization of the transformation parameters together with selection of an appropriate transformation can lead to significant improvements in model fitting during automated gating that compare favourably to results obtained for the untransformed data. Furthermore, the estimated transformation parameters compare favourable with the true transformation parameters (Additional File 3), demonstrating that our assumption of a global multivariate Gaussian distribution is acceptable for the purpose of transformation.

Following extraction of discovered cell populations and metaclustering, we observed the lowest intra-cluster variability for the optimized generalized arcsinh and optimized biexponential transformations, followed by the baseline of no data transformation, indicating that parameter optimization can reduce inter-sample variability and aid population matching (Figure 2c). The largest inter-sample variability following meta-clustering was observed for the optimized generalized Box-Cox, default generalized arcsinh, and optimized linlog transformations (Figure 2c). Selected metaclusters obtained from default and parameter-optimized transformations demonstrate that parameter optimized transformations can lead to better population identification in the automated gating step, and consequently lower variability metaclusters (Figure 2d).

### Software and Availability

We have implemented parameter-optimization routines for the biexponential, linlog, generalized arcsinh, and generalized Box-Cox transformations in the R package, *flowTrans*. This package integrates with the existing FCM data analysis tools in BioConductor and uses existing data structures data manipulation paradigms from the *flowCore *framework [22,25]. The package is freely available at http://www.bioconductor.org/packages/release/bioc/html/flowTrans.html.

## Discussion

Ideally, all cell subpopulations in an FCM sample would be well-separated to facilitate gating. In practice, this is rarely, if ever the case. Real flow data typically consists of a mixture of complicated distributions that are asymmetric, frequently overlapping, with cell populations whose variances are dependent on their mean fluorescence intensities. A variety of automated gating algorithms have been proposed to identify distinct cell sub-populations [1,6-10]. Most of these automated approaches function under the assumption that the data has been transformed prior to the automated gating step. Others even include data transformation as part of the gating procedure [6,8,10]. The global data transformation step treats all cells and cell subpopulations equally, and hides an implicit assumption of a common error model across all cells and cell subpopulations. The problem is that we do not know what the correct error model is for FCM data, and so it is simply ignored. In this paper, our approach to parameter optimization attempts to make this assumption more explicit. We assume that, on the transformed scale, the global data distribution can be approximated by a multivariate Gaussian distribution. Although the data are clearly not multivariate normal, as typical FCM data are multimodal and best represent by a mixture model, we transform the data towards normality to make the distribution more symmetric, mitigate the impact of outliers, and to have an objective criterion to use for estimating transformation parameters. Our simulation study shows that our assumption of a common Gaussian distribution allows us to obtain reasonable estimates of the optimal transformation parameters even when the Gaussian assumption is violated. Only subsequently do we take on the task accurately modelling the data using a model-based automated gating algorithm. We leave the work of fitting a mixture model to resulting transformed data to the automated gating algorithm.

We have examined the impact of the choice of transformation and its corresponding parameters on the automated gating procedure. Transforming the data towards normality reduces the influence of outlier events and our simulations have shown that optimizing transformation parameters in this way can improve gating and cell subpopulation discovery when compared to applying a naive transformation with default parameter values. Optimization of the parameters leads to lower misclassification rates, improved cell population identification, lower inter-sample variability and fewer outliers than blindly applying default transformation parameters. In our simulations, data were transformed with the biexponential function using randomly chosen parameters. Only parameter-optimized transformations from the same family (optimized generalized arcsinh and optimized biexponential) regenerated a data distribution similar to the untransformed data, as exhibited by comparable gating misclassification rates (Figure 2b). The optimized linlog and generalized Box-Cox transformations did not have the flexibility to transform the data back to the original distribution, leading to biases in the automated gating step. Although the differences in misclassification rate are only a few percent, they could introduce significant variation on downstream analysis, especially in large-sample situations, where many data sets need to be analyzed in an automated manner.

Our analysis of a subset of real-world flow cytometry data set derived from lymph node biopsies of individuals with follicular lymphoma demonstrated similar results to those obtained using simulated data. Optimizing transformation parameters in order to make the transformed FCM data more normal-like can improve data visualization and cell subpopulation identification in certain cases. However the improvement in performance is data- dependent, and it is unclear how to determine in advance which samples benefit from such an approach as compared to applying a standard FCM transformation. Addressing this problem is the subject of future work. In the cases examined in this study, transformation of the scatter dimensions via a parameter-optimized generalized arcsinh or biexponential transformation generally improves cell subpopulation visualization (Figure 3). Therefore, in situations where scatter data are to be gated manually we argue in favour of such an approach. However, we note that under an automated gating scheme, the differences between transformations are marginal on the scale of the data, and the benefits are minimal, particularly when FSC and SSC cell populations are well defined. This is in accordance with what is typically seen in FCM data analysis [14].

Cell subpopulation identification in the fluorescence channels benefits more from parameter- optimized transformation than in the scatter channels. However, again the choice of transformation is data-dependent. While the greatest improvement in metacluster variability was observed for the parameter-optimized generalized arcsinh transformation in the case of scatter channels, here the greatest improvement is observed for the parameter-optimized biexponential transformation and parameter-optimized generalized Box-Cox transformation. The intra-metacluster variation for these transforms is lower than for the log transformation, but only marginally lower than for the default- parameter biexponential transformation. Despite this, closer examination of the metaclusters generated from the default biexponential transformed data shows that one of the CD3-/CD5+/CD19+ cell subpopulations was not captured by the CD3-/CD5+/CD19+ metacluster. Additionally, the default biexponential transformed data leads to seven metaclusters, compared to six metaclusters obtained for the optimized biexponential transformed data. These additional metaclusters capture outlier cell populations which are not of interest in this particular experiment. In general, the metaclusters representing the primary cell subpopulations in this data all capture representatives from each of the ten samples. The predominant source of intra-metacluster variability is derived from the metaclusters corresponding to outlier cell populations. Therefore, in situations where an automated gating and analysis approach is undertaken, we recommend applying a parameter-optimized data transformation such as the optimized biexponential, rather than the default biexponential, since it has the potential to improve cell subpopulation discovery and matching across multiple samples. This is likely to be a greater concern in large-sample situations than when a small number of samples are to be analyzed manually.

Our metaclustering approach has been designed to work specifically for data gated using the *flowClust/flowMerge *algorithms. The constraint limiting each metacluster to one cell population per sample is predicated on the assumption that the gating algorithm (*flowMerge *in our case) represents each distinct cell population by a unique mixture component. Generally speaking, this is a safe assumption in the case of the *flowMerge *algorithm, which has been designed to identify and represent distinct cell populations by unique mixture components [4]. However, the metaclustering algorithm would have to be modified if the gating algorithm were changed.

We explored alternative approaches to parameter estimation that included preselection of a sub-population of events and optimizing transformation parameters with respect to the subpopulation. How-ever, this approach proved ineffective, since preselection effectively removed outlier events. It is these outliers that cause problems for downstream automated gating approaches, and thus should be considered in the transformation step. The decision to transform the data towards a multivariate Gaussian distribution is supported by our ability to accurately recover the true transformation parameters in our simulation study, even though the original data distribution is clearly derived from a multivariate mixture model. Our approach could be extended in a number of ways analogous to *flowClust*, either by embedding the transformations within the gating step, thus selecting transformation parameters maximize the likelihood of a K-component mixture rather than a single component density, or by modelling the data with a more robust distribution such as the multivariate-*t*. This approach could have the benefit of generating less variable parameter estimates, though likely at the expense of computation time.

## Conclusions

Although the idea of optimizing transformations for FCM data is not new, to date, there has been no systematic comparison of FCM data transformations examining their performance in an automated data analysis setting [6]. We have developed criteria for optimizing the parameters of transformations commonly used for preprocessing and visualization of FCM data, designed to transform the data towards a more multivariate normal and symmetric global data distribution. We have shown that these parameter-optimized transformations can improve data visualization, population discovery, and metaclustering, relative to their default-parameter counterparts in certain sample-specific cases. Parameter optimization of the generalized Box-Cox transform has been previously implemented within an automated gating framework implementing a mixture modelling approach (*flowClust*) [6]. While it would be of interest to implement a similar strategy for the other commonly used flow cytometry data transformations, our goal here has been to examine the influence of global transformation on the automated gating step of high throughput FCM analysis. Our software allows the user to quickly examine the effects of different parameter-optimized transformations on the data, and defer the computation-intensive gating step to downstream analysis. Our findings showed that the optimized generalized arcsinh transformation had the lowest intra-sample variability between populations for the scatter channels. However, substantive differences in variation were subtle and rare across the different transformations in the scatter channels. In contrast, the optimized biexponential transform had the lowest variability for the fluorescence channels. This transformation is in accordance with current best practices. Therefore we would recommend, in the absence of additional knowledge to suggest a given data transformation, the parameter-optimized versions of the biexponential transform for fluorescence channel data, over the default-parameter counterpart. In situations where many samples need to be processed in an automated manner, the parameter-optimized generalized arcsinh may be a better choice over the current standard practice of not transforming the scatter data, thus reaping the benefits of occasional improvements in the automated gating step. Due to its simplicity, our optimization algorithm could be readily implemented in other widely available tools such as *FlowJo *and *WinList*, and indeed, efforts are currently underway to tie *FlowJo *more closely to the *R *computing environment [11].

## Authors' contributions

GF designed experiments, performed analysis, and contributed to manuscript preparation. RG designed experiments and contributed to manuscript preparation. JMP contributed to manuscript preparation. AW contributed the clinical FCM data.

## Supplementary Material

Additional file 1 **Examples of common transformations with typical parameters**. Examples of the generalized Box-Cox (blue, *θ *= 0.158), linlog (red, *θ *= 56.9), biexponential (black, *a *= 0.49, *b *= 0.99, *c *= 1, *d *= 0.01, *f *= 0, *w *= 2.3), and generalized arcsinh (green, *a *= 1, *b *= 0.052) transformations using common parameter values. The chosen parameters are selected from an optimized fit of each transformation to FSC vs SSC follicular lymphoma data. Click here for file

Additional file 2 **The biexponential transformation is weakly identifiable**. The biexponential transformation with full parameterization is weakly identifiable. a) A bivariate normal distribution on the original scale. b) Original data transformed with the inverse-biexponential using parameters *a *= 1, *b *= 1, *c *= 1, *d *= 1 10^-10^, *f *= 0, *w *= 0. c) Inverse-transformed data transformed with the biexponential using the true parameters *a *= 1; *b *= 1, *c *= 1, *d *= 1 × 10^-10^, *f *= 0, *w *= 0. d) Inverse-transformed data transformed with the biexponential using alternate parameters *c' *= 1 × 10^-10^, *d' *= 1 × 10^-10^, *f' *= -1. When parameters *c' *and *d' *are near zero, if *f' *≈ *c *and *d' *is near zero, the two transformations are virtually indistinguishable. Click here for file

Additional file 3 **Residuals of estimated biexponential parameters**. Boxplots showing the difference between true and estimated biexponential parameters on ten simulated data sets. Although the optimized estimates are variable for parameters b, d, the misclassification rates of fitted models demonstrate that this bias doesn't negatively impact the subsequent gating. Click here for file
